# *Erwinia billingiae* as Unusual Cause of Septic Arthritis, France, 2017

**DOI:** 10.3201/eid2508.181073

**Published:** 2019-08

**Authors:** Isabelle Bonnet, Baptiste Bozzi, Eric Fourniols, Stéphane Mitrovic, Olivia Soulier-Escrihuela, Florence Brossier, Wladimir Sougakoff, Jérôme Robert, Stéphane Jauréguiberry, Alexandra Aubry, on behalf of the Pitié-Salpêtrière Infection Ostéo-articulaire group

**Affiliations:** Assistance Publique–Hôpitaux de Paris, Hôpitaux Universitaires Pitié Salpêtrière–Charles Foix, Paris, France (I. Bonnet, B. Bozzi, E. Fourniols, S. Mitrovic, O. Soulier-Escrihuela, F. Brossier, W. Sougakoff, J. Robert, S. Jauréguiberry, A. Aubry);; Sorbonne Université, Cimi-Paris, U1135, Paris (I. Bonnet, F. Brossier, W. Sougakoff, J. Robert, A. Aubry)

**Keywords:** knee septic arthritis, *Erwinia billingiae*, *Enterobacteriaceae*, plant, palm tree, 16S rRNA, injury, bacteria, MALDI-TOF mass spectrometry, MALDI-TOF, literature review, case study, France

## Abstract

In 2017 in France, we treated a patient with knee septic arthritis caused by *Erwinia billingiae* after trauma involving a palm tree. This rare pathogen could only be identified through 16S rRNA gene sequencing. For bacterial infections after injuries with plants, 16S rRNA gene sequencing might be required for species identification.

The prevalence of acute septic arthritis in Western Europe is ≈4–10 cases/100,000 inhabitants (*1*). We report a case of posttraumatic knee septic arthritis in an immunocompetent patient in France that was caused by *Erwinia billingiae*, a gram-negative environmental bacterium of the family *Enterobacteriaceae*. We also review the characteristics of *Erwinia* species and infections.

On April 9, 2017, a 65-year-old man with an unremarkable medical history was admitted to an emergency unit in Nice, southern France, for painful right knee swelling that occurred a few hours after a Phoenix palm tree needle pierced the area. The foreign body was partly removed, and the wound was sutured. The patient was discharged without any knee pain and given a prescription for amoxicillin/clavulanic acid (1 g 3×/d for 6 d).

On April 22, the patient was admitted to the emergency unit of our hospital in Paris because of sudden right knee pain and fever. Synovial fluid collected by knee puncture the day of his admission to the orthopedic unit (April 23) contained 118 × 10^9^ leukocytes/L, consisting of 64% polymorphonuclear cells, 33% lymphocytes, and 3% other leukocytes; no microorganism could be identified after Gram staining and cultures. A second knee puncture was performed 3 days after admission, and gram-negative rods grew within 2 days solely within the anaerobic blood culture vial (BacT/ALERT SN; bioMérieux, https://www.biomerieux.com). Subcultures of the blood culture vial were positive after 24 hours of incubation at 37°C on blood agar (Trypticase Soy agar + 5% horse blood and Mueller Hinton 2 agar + 5% sheep blood; bioMérieux) and Drigalski agar (BD, https://www.bd.com) under aerobic conditions and chocolate agar (BD) under microaerobic conditions.

Matrix-assisted laser desorption/ionization time-of-flight (MALDI-TOF) mass spectrometry (Bruker Daltonik, https://www.bruker.com) was performed on colonies and failed to correctly identify the species. Therefore, we performed species identification by 16S rRNA amplification and sequencing with primers RNA-S (16S, 5′-AGAGTTTGATCCTGGYTCAG-3′) and RNA-AS (16AS, 5′-CTTTACGCCCARTAAWTCCG-3′) at a hybridization temperature of 52°C. We amplified a 521-bp sequence that matched the *E. billingiae* genome of 2 isolates with 99.4% similarity (GenBank accession nos. JQ929658 and JN175337). Other closely related species displayed lower similarities: *Pantoea rwandensis* (99.0%), *Erwinia persicina* (98.9%), *Pantoea coffeiphila* (98.7%), *Erwinia tasmaniensis* (98.5%), and *Erwinia aphidicola* (98.3%). Following guidelines of the Antibiogram Committee of the French Society for Microbiology (https://www.sfm-microbiologie.org/2019/01/07/casfm-eucast-2019), we tested the *E*. *billingiae* isolate with the antimicrobial drugs recommended for *Enterobacteriaceae*; the isolate was susceptible to all these drugs, including ampicillin.

Because of the lack of clinical improvement, the joint was washed on day 6 after admission. After this intervention, an empiric antimicrobial drug treatment was started with amoxicillin/clavulanic acid (2 g 3×/d intravenously). Once results of drug susceptibility testing became available (i.e., 10 days after admission), his treatment was switched to cefotaxime (2 g 3×/d intravenously) and ciprofloxacin (500 mg 2×/d orally for 8 d), followed by ciprofloxacin (500 mg 2×/d alone for 38 additional days). Total duration of treatment was 45 days. The clinical evolution of this patient was favorable; he fully recovered and had no relapses up to 1 year after treatment completion.

In the past, some members of the *Erwinia* genus were reassigned to the genera *Enterobacter* or *Pantoea*. *Erwinia* spp. are ubiquitous in the environment, especially in water ecosystems and soils. Plant-associated *Erwinia* species comprise epiphytic nonpathogenic (i.e., *E. billingiae* and *E. tasmaniensis*) and pathogenic (i.e., *E. amylovora* and *E. pyrifoliae*) species. The MALDI-TOF mass spectrometry system failed to identify the bacterium, even though *E. billingiae* is contained in the database for either method used (direct deposit or on-plate formic acid treatment). Future expansion of the database with more spectra will likely improve the performance of the MALDI-TOF mass spectrometry system for *E. billingiae* identification. Indeed, the database contains fewer spectra of *E. billingiae* (n = 4) than those of frequently encountered species in medical microbiological laboratories, such as *Escherichia coli* (n = 14) and *Staphylococcus aureus* (n = 10).

To further investigate *Erwinia* infections in humans, we reviewed reports available in PubMed published during 1967–2017 written in English by using the keywords “*Erwinia*” and “infection” (Table). Among the 17 cases reported, the sites of infection were diverse, and most (53%, 9/17) cases occurred after a direct inoculation during an injury with a plant (Table). We found no reports of osteoarticular infections with *Erwinia*; the only other *E. billingiae* case reported was a dermohypodermitis (Table). In that case, as in the case we report here, an injury with a plant was reported.

**Table:** Case reports from the literature of infection caused by *Erwinia* spp.*

Patient age, y/sex	Type of infection	Inoculated	Published (actual) species name	Identification method†	Antimicrobial drug; treatment duration	Surgery	Clinical evolution	Ref
65/F	SSTI	Yes	*Erwinia* sp.	Biochemical	Penicillin, then penicillin and sulfisoxazole; NA	Yes	Recovered	(*2*)
Adult/F	Peritoneal dialysis fluid infection	No	*Erwinia* strains of the lathyri-herbicola group	Biochemical	NA; NA	No	NA	(*3*)
Adult/F	SSTI	Yes	*Erwinia* strains of the lathyri-herbicola group	Biochemical	Chloramphenicol; NA	No	Recovered	(*3*)
Adult/M	SSTI	Yes	*Erwinia* strains of the lathyri-herbicola group	Biochemical	Ampicillin; NA	No	Recovered	(*3*)
Adult/M	SSTI	Yes	*Erwinia* strains of the lathyri-herbicola group	Biochemical	Penicillin; NA	No	Recovered	(*3*)
Adult/M	Brain abscess	No	*Erwinia* strains of the lathyri-herbicola group	Biochemical	NA; NA	Yes	NA	(*3*)
17/F	Bacteremia	Yes	*Erwinia herbicola*; (*Pantoea agglomerans*)	Biochemical	Streptomycin and penicillin; NA	No	Recovered	(*4*)
17/M	Bacteremia	Yes	*E. herbicola *(*P. agglomerans*)	Biochemical	Cephalothin; NA	No	Recovered	(*4*)
28/M	Bacteremia	Yes	*Erwinia* sp.	Biochemical	Ampicillin then ampicillin and kanamycin; NA	No	Recovered	(*4*)
57/M	Brain abscess	No	*Erwinia* sp.	Biochemical	Penicillin and streptomycin, then ampicillin, then chloramphenicol, then gentamicin; NA	Yes	Recovered	(*5*)
70/M	Endophtalmitis	Yes	*E. herbicola *(*P. gglomerans*)	Biochemical	Cefazolin and gentamicin; 37 d until surgery (NA after surgery)	Yes	Recovered	(*6*)
66/F	UTI	No	*E. herbicola *(*P. agglomerans*)	Biochemical	NA; NA	No	Died	(*7*)
69/F	UTI	No	*E. herbicola *(*P. agglomerans*)	Biochemical	NA; NA	No	Recovered	(*7*)
62/F	UTI	No	*E. herbicola *(*P. agglomerans*)	Biochemical	NA; NA	No	Recovered	(*7*)
46/M	Endocarditis	No	*E. herbicola *(*P. agglomerans*)	Biochemical	Cefotaxime and netilmicin; 6 weeks	No	Recovered	(*8*)
79/F	Cervical lymphadenitis	No	*Erwinia tasmaniensis *(*E. tasmaniensis*	16S rRNA‡	Ciprofloxacin; 2 weeks	Yes	Recovered	(*9*)
40/M	Dermohypodermitis	Yes	*Erwinia billingiae *(*E. billingiae*)	NA	Ciprofloxacin; 14 d	No	Recovered	(*10*)

*NA, not available; ref, reference; SSTI, skin and soft tissue infection; UTI, urinary tract infection. †Biochemical testing included Kligler iron agar (assess slant, butt, H_2_S production), tests for carbohydrate fermentation (adonitol, fructose, galactose, glucose, inositol, lactose, maltose, mannitol, mannose, raffinose, rhamnose, salicin, sorbitol, sucrose, xylose), ONPG (ortho-nitrophenyl-galactosidez) test, gluconate test, gelatin hydrolysis test, tests for nitrate reduction and N_2_ production, indole test, methyl red test, Voges–Proskauer test, casein hydrolysis test, citrate utilization test, urease test, catalase test, oxidase test, arginine dihydrolase test, lysine decarboxylase test, ornithine decarboxylase test, lipase test, amylase test, pectinase test, deoxyribonuclease test, lecithinase test, salinity tests (2.5% NaCl, 10.0% NaCl [pH 5.6]), Tetrazolium-Formazan test (TTC [triphenyl tetrazolium chloride]), cetrimide selection agar, tyrosinase test, and tests for carbohydrate assimilation (glucose, acetate, lactate, succinate). ‡*E. tasmaniensis* (98.9%), *E. toletana* (98.8%), and *E. billingiae* (98.1%) (EzTaxon Database, https://everipedia.org/wiki/lang_en/EzTaxon_Database).

This case report illustrates the importance of the methods used for bacterial identification to correctly diagnose such infections. Biochemical methods (*2*–*8*) and MALDI-TOF mass spectrometry (as done in our investigation) could result in misidentification. This report highlights the usefulness of analyzing MALDI-TOF mass spectrometry scores before assigning a species identity and sequencing the 16S RNA gene for bacteria not identifiable by conventional methods.
